# Exploratory analyses of meal‐induced heartburn identify distinct clinical phenotypes of gastroesophageal reflux disease

**DOI:** 10.14814/phy2.70469

**Published:** 2025-07-22

**Authors:** Jerry D. Gardner, George Triadafilopoulos

**Affiliations:** ^1^ Science for Organizations Mill Valley California USA; ^2^ Division of Gastroenterology and Hepatology Stanford University School of Medicine Redwood City California USA; ^3^ Department of Gastroenterology, Hepatology and Nutrition UT MD Anderson Cancer Center Conroe Texas USA

**Keywords:** gastroesophageal reflux disease, heartburn, phenotypes

## Abstract

Symptoms in symptomatic GERD patients result from esophageal acid exposure and are treated with agents that inhibit gastric acid secretion. We analyzed data from a clinical trial evaluating the effect of ranitidine plus antacid on heartburn severity in GERD patients. After 26 subjects ingested a standard meal and received either placebo or ranitidine‐antacid, heartburn severity (assessed via a visual analog scale), esophageal pH, and gastric pH were measured at 15‐min intervals. Two phenotypes emerged based on heartburn severity patterns: “Persistent Heartburn” (PH), characterized by sustained high severity, and “Non‐persistent Heartburn” (NPH), where severity peaked and then declined. PH subjects had similar gastric acidity but higher overall heartburn severity and lower esophageal acid exposure than NPH subjects. Both phenotypes exhibited esophageal hyperalgesia, with significant heartburn even at esophageal pH above 4.0, and hyperalgesia was more pronounced in PH subjects. These findings suggest that esophageal sensitivity, rather than acid exposure alone, contributes to symptom severity. The differing responses to placebo and ranitidine‐antacid highlight potential mechanisms underlying treatment failure in some GERD patients, emphasizing the need for tailored therapeutic approaches.

## 1 INTRODUCTION

Using data from previous clinical studies can offer valuable insights, particularly when exploring new hypotheses. The present analyses use data from a proof‐of‐principle clinical trial examining the effects of ranitidine plus antacid on heartburn severity in symptomatic GERD subjects (Robinson et al., 2001). As often happens in post hoc analyses, the trial's design may differ from an ideal prospective study, but these differences do not undermine the findings and may offer additional context. According to Tukey (Tukey, 1977), exploratory data analyses help “see what happened,” offering provisional insights into data patterns and relationships.

Most previous reports that have focused on GERD symptoms have not measured esophageal pH (Allgood et al., 2005; Laine et al., 2024). On the other hand, most reports that have measured esophageal pH, and possibly gastric pH and esophageal impedance as well, have focused on esophageal acid exposure time, relationships between symptom frequency and reflux episodes, and esophageal pH when symptoms and reflux episodes occur together (Ghisa et al., 2020; Gyawali et al., 2018). Only a few studies have examined esophageal acid exposure that is associated with individual symptoms (Gardner, 2022a, 2022b).

In the present exploratory analyses, we investigate how meal‐induced heartburn in GERD patients can reveal relationships among heartburn severity, esophageal acid exposure, and gastric acid concentrations, as well as how these relationships shift with different treatments.

## 2 SUBJECTS AND METHODS

Data from a previous analysis (Robinson et al., 2001) were re‐examined. This study was approved by and conducted in compliance with Good Clinical Practices as supervised by the Western Institutional Review Board, Olympia, WA. All subjects enrolled in this study gave written informed consent.

The clinical trial that generated the data used for the present analyses was a proof‐of‐principle study intended to examine the potential of a combination product containing ranitidine plus an antacid to be approved by the US FDA as a nonprescription (over‐the‐counter) product to relieve heartburn caused by indigestion or sour stomach. The trial was designed to mimic the settings in which subjects might use the product.

The study included 26 subjects (10 men and 16 women) with heartburn that was responsive to antacids occurring more than four times per week for at least 2 months. Subjects were not tested for *H. pylori* infection and did not undergo upper gastrointestinal endoscopy. Subjects were free of significant gastrointestinal disorders aside from heartburn or GERD and had not taken medications affecting gastric acid secretion or motility within 7 days before study entry, or investigational drugs within 30 days prior to study entry.

The randomized, four‐way crossover study assessed the effects of four treatments on heartburn severity, gastric pH, and esophageal pH: Placebo ranitidine; ranitidine (75 mg); placebo ranitidine + chewable antacid (420 mg calcium carbonate); ranitidine (75 mg) + chewable antacid. Ranitidine alone and antacid alone were included because of the requirement by the US Food and Drug Administration that the effect of a combination product must be significantly greater than the effect of each constituent alone.

Ranitidine and placebo‐ranitidine each were taken with 120 mL water; the antacid was chewed and swallowed. Blinding was applied to ranitidine and placebo‐ranitidine but not to the antacid. Subjects chose the timing of treatment to relieve heartburn. Washout periods of 7–10 days were observed between treatments.

Heartburn severity was measured every 15 min for 4.5 h beginning at the end of the meal using a 100‐mm visual analog scale (VAS). Gastric and esophageal pH values were recorded every 4 s for 1 h premeal, during the meal, and 4.5 h post‐meal. The gastric pH electrode was placed 10 cm below, and the esophageal pH electrode 5 cm above the manometrically defined upper border of the lower esophageal sphincter. The meal consisted of sausage, egg, cheese biscuit, 30 g raw onion, 250 mL chocolate milk, and a peppermint patty, all consumed within 30 min. Subjects could eat up to three servings of any meal component, maintaining consistency across treatments. Meal size was quantified by homogenizing the total meal and titrating it to pH 2.0 with 0.1 N HCl.

## 3 ANALYSIS

Because our present analyses focused on effects of an active treatment that reduced heartburn severity, we considered only values for VAS, esophageal pH, and gastric pH in subjects treated with placebo or ranitidine plus antacid (ranitidine‐antacid). Mean acid concentrations were calculated for 15‐min intervals beginning at the end of the meal. Thus, each value of VAS was accompanied by values for mean esophageal acid concentration and mean gastric acid concentration over the 15‐min interval that preceded the VAS value.

Statistical analyses and curve fitting were performed using GraphPad Prism 10.4.0. *p* values were not adjusted for multiple comparisons, as the analyses were exploratory.

## 4 RESULTS

**FIGURE 1:**
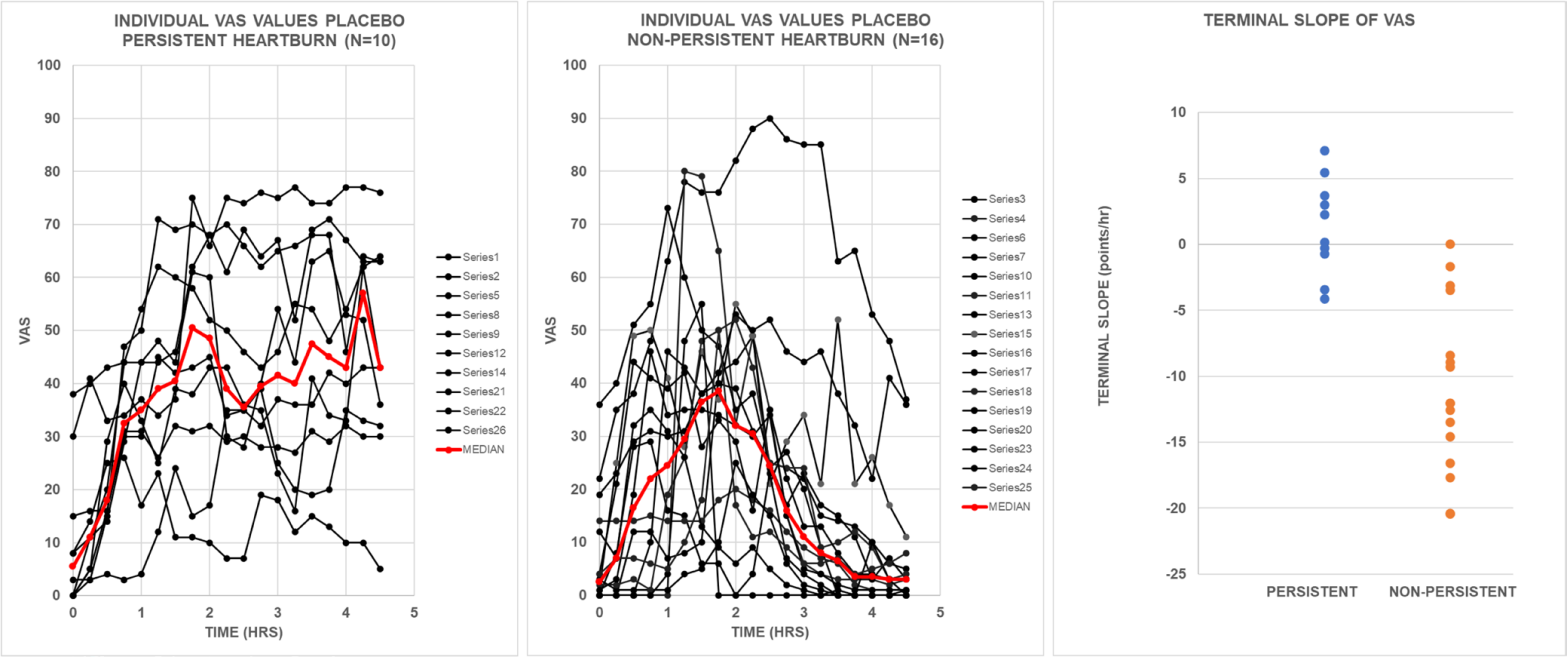
Values from the visual analog scale (VAS) for individual subjects treated with placebo. Left panel gives values for the VAS from each of 10 individual subjects that had a slope of the VAS‐time curve beginning at 2.0 h that was not negative and not significantly different from zero (*p* > 0.05) by an *F*‐test. MIDDLE PANEL gives values for the VAS from each of 16 individual subjects that had a slope of the VAS‐time curve beginning at 2.0 h that was negative and significantly different from zero (*p* < 0.05) by an *F*‐test. The solid red line in each panel gives the median value at each 15‐min time point. Right panel gives values for slope of the VAS‐time curve beginning at 2.0 h. The slopes from four subjects with persistent heartburn (PH) that were negative and that from the one subject with nonpersistent heartburn (NPH) that was positive were not significantly different from zero.

Figure 1 demonstrates that all 26 study participants experienced heartburn after consuming a meal commonly eaten by millions of Americans. It also highlights two distinct patterns in the time course of VAS values. For each participant, the least‐squares slope of VAS values was calculated for the final 2.5 h of the recording period. In Figure 1 left, 10 subjects had a slope that was positive or not significantly different from zero. Median VAS values for these individuals were categorized as “Persistent Heartburn” (PH). In Figure 1 middle, 16 subjects had a negative slope that was significantly different from zero. Median VAS values for these individuals were categorized as “Non‐Persistent Heartburn” (NPH). In Figure 1 right, each slope from subjects with NPH was negative and significantly different from zero (*p* < 0.05) by an *F*‐test. No slope from subjects with PH was both negative and significantly different from zero. We examined the possibility that the differences in slopes reflected a period effect and found that the period numbers from subjects with PH were not significantly different from those from subjects with NPH (*p* = 0.404 by Mann–Whitney test). Thus, values from the time course of heartburn severity following the end of a standard meal from a subject with symptomatic GERD can be used to assign the subject to one of the two clinical phenotypes.

To investigate whether differences in meal size contributed to the observed patterns in Figure 1, the amount of hydrochloric acid (HCl) needed to titrate homogenized meal samples to pH 2 was analyzed. The HCl requirement ranged from 94 mmol to 174 mmol across both groups. The mean HCl requirement was 142 mmol for subjects with PH and 132 mmol for those with NPH. This difference was not statistically significant (*p* = 0.4132, Unpaired *t*‐test).

Since esophageal acid is generally believed to be the cause of heartburn, we examined the time courses for heartburn severity and esophageal acid concentration with placebo and ranitidine‐antacid in subjects with PH or NPH.

**FIGURE 2:**
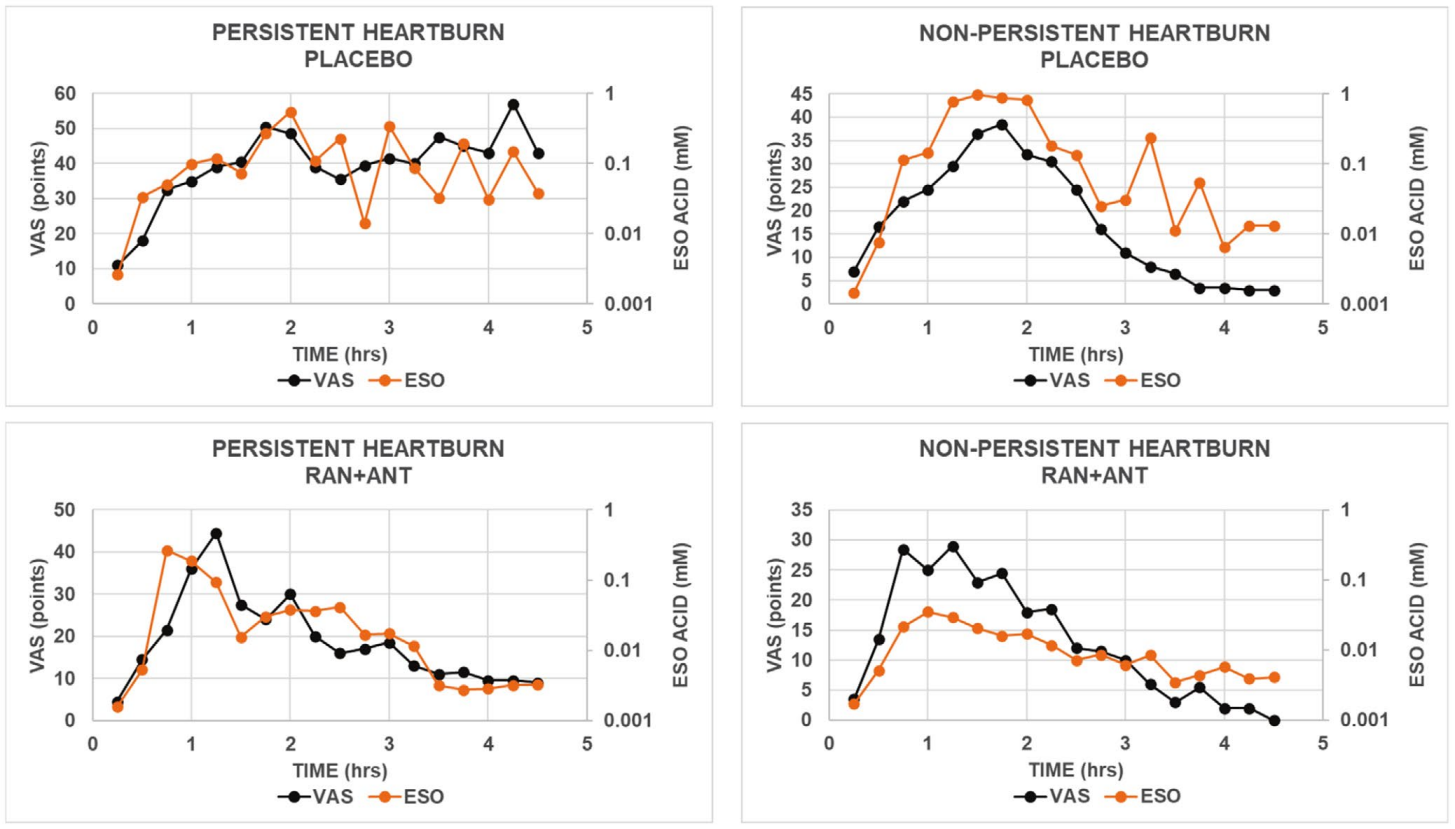
Values for heartburn severity and mean esophageal acid concentration with placebo or ranitidine‐antacid were calculated for each 0.25‐h interval beginning at the end of ingestion of the standard meal. Treatments were administered 1 h after the end of the meal. Solid symbols are median values for VAS or acid concentration for subjects with PH (*N* = 10) or NPH (*N* = 16). ANT, antacid; ESO, esophageal; RAN, ranitidine; VAS, visual analog scale.

In Figure 2, all panels show that the configuration of the time course for esophageal acid was similar to that for VAS. In the top left panel of Figure 2, the configurations of the time courses with placebo for both VAS and esophageal acid increased to a plateau at approximately 2 h and remained elevated for the duration of the assessment period. In the remaining 3 panels, the configurations of the time courses for both VAS and esophageal acid increased to a maximum at 1–2 h and then decreased toward the baseline during the remainder of the assessment period.

In the top 2 panels of Figure 2 showing results with placebo, the sum of the values for VAS in subjects with PH was higher than that in subjects with NPH, while the sum of the values for esophageal acid concentration in subjects with PH was lower than that in subjects with NPH. These results indicate that in terms of overall heartburn severity, esophageal acid sensitivity in subjects with PH was higher than that in subjects with NPH.

The top panels in Figure 2 also show that with placebo, converting esophageal acid concentration (mM) to pH indicates that in subjects with PH, esophageal pH ranged from 3.3 to 5.6 with 56% of values being above pH 4, and that in subjects with NPH, esophageal pH ranged from 3.0 to 5.8 with 50% of values being above pH 4. Thus, a majority of median values for heartburn severity were associated with values of esophageal acid concentration above pH 4.0, indicating esophageal hyperalgesia.

Finally, the top panels in Figure 2 show that the terminal portions of the time courses for esophageal acid concentrations show substantial oscillations that are not accompanied by changes in heartburn severity of comparable magnitude. In subjects with PH, these oscillations began at hour 2, and in subjects with NPH, they began at hour 3. In both groups of subjects, these oscillations tend to occur with low esophageal acid concentrations that are above pH 4. Even with the oscillations, however, the time course for esophageal acid concentrations corresponded to the time course for heartburn severity.

Ranitidine‐antacid decreased both heartburn severity and esophageal acid concentrations in subjects with PH and in those with NPH (Figure 2). That is, in subjects with PH or with NPH, the sum of the values VAS and that for esophageal acid concentrations was lower with ranitidine‐antacid than with placebo. The bottom panels in Figure 2 also show that with ranitidine‐antacid, total values for both VAS and esophageal acid concentration were higher in subjects with PH than in subjects with NPH.

The bottom panels in Figure 2 show that with ranitidine‐antacid, there were relatively small oscillations of values for esophageal acid concentration and corresponding values for heartburn severity in both phenotypes. These oscillations will become clearer when values of heartburn severity are plotted as functions of corresponding values of esophageal acid concentrations in Figure 4.

The bottom panels in Figure 2 also show that with ranitidine‐antacid, converting esophageal acid concentration (mM) to pH indicates that in subjects with PH, esophageal pH ranged from 3.6 to 5.8 with 89% of values being above pH 4, and that in subjects with NPH, esophageal pH ranged from 4.4 to 5.8 with 100% of values being above pH 4. Thus, all or nearly all median values for heartburn severity were associated with values of esophageal acid concentration above pH 4. In addition, in both groups of subjects, the proportional decrease in esophageal acid concentration with ranitidine‐antacid was substantially greater than the corresponding proportional decrease in heartburn severity, reflecting esophageal hyperalgesia.

The results in Figure 2 indicate that esophageal hyperalgesia is an essential feature of symptomatic GERD that can limit the effect of reduced esophageal acid concentration on heartburn severity that occurs following a meal. This hyperalgesia can also limit the magnitude of the change in heartburn severity that occurs with oscillations of esophageal acid concentrations.

Since gastric acid is generally believed to be the source of esophageal acid, we examined the time courses for gastric acid concentration and esophageal acid concentration with placebo and ranitidine‐antacid in subjects with PH or NPH.

**FIGURE 3:**
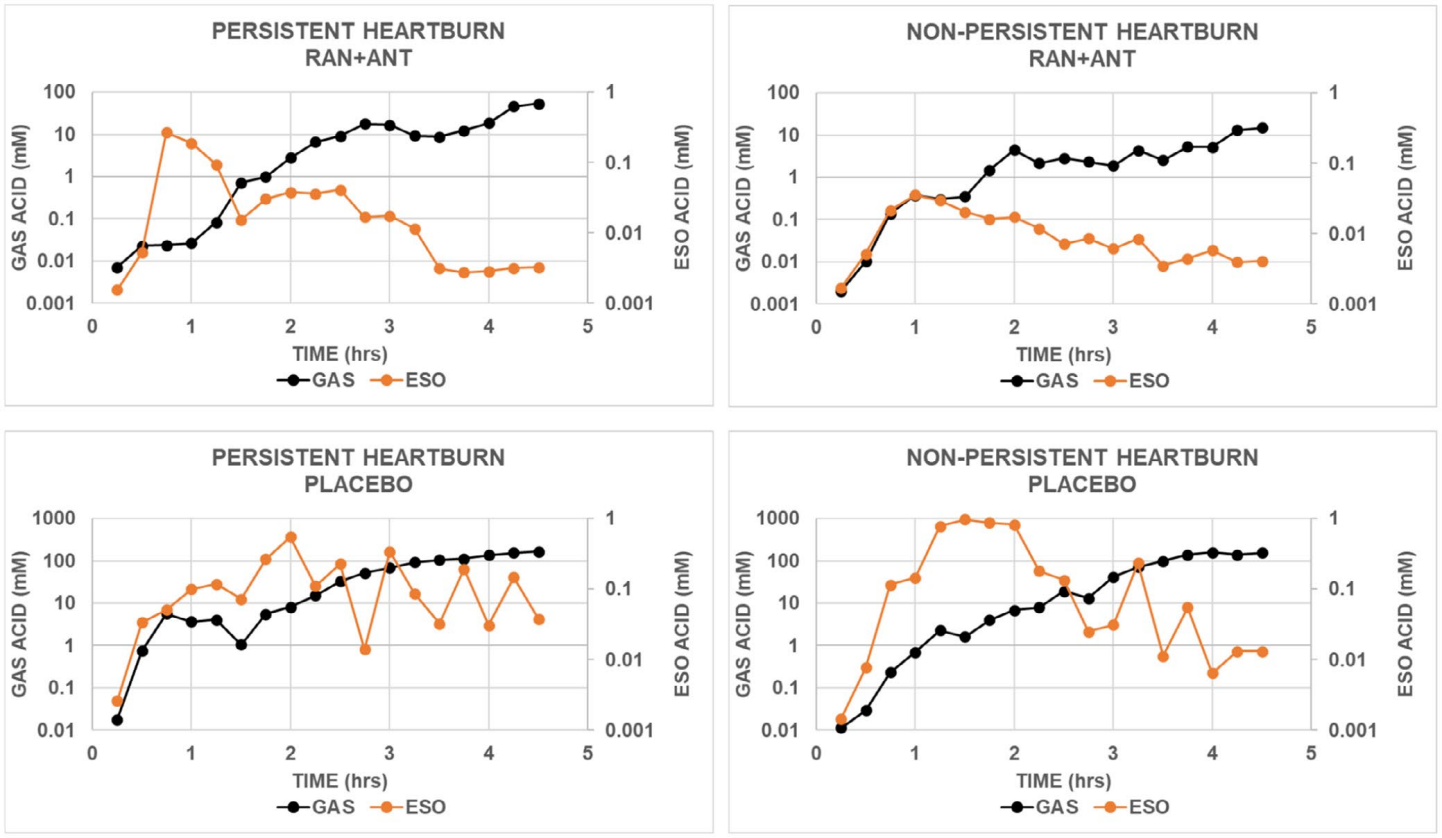
Values for mean gastric acid concentration and mean esophageal acid concentration with placebo or ranitidine‐antacid were calculated for each 0.25‐h interval beginning at the end of ingestion of the standard meal. Treatments were administered 1 h after the end of the meal. Solid symbols are median values for acid concentration for subjects with PH (*N* = 10) or NPH (*N* = 16). ANT, antacid; ESO, esophageal; GAS, gastric; RAN, ranitidine; VAS, visual analog scale.

In Figure 3, the data for esophageal acid concentrations are the same as those shown previously in Figure 2. All panels in Figure 3 show a similar configuration of the time course for gastric acid concentration in that it increased progressively during the 4.5‐h assessment period. No time course for esophageal acid concentration corresponded to that for the accompanying gastric acid concentration. In subjects with PH treated with placebo, esophageal acid concentrations increased to a plateau and remained elevated, while in subjects with PH treated with ranitidine‐antacid, and in subjects with NPH treated with placebo or ranitidine‐antacid, esophageal acid concentrations increased to a peak and then decreased.

The top panels in Figure 3 show that for the oscillations in esophageal acid concentrations in subjects treated with placebo, there was no corresponding change in gastric acid concentrations. Thus, some presently unidentified feature of the post‐meal period probably influences the passage of acid from the stomach into the esophagus.

The sum of the values for gastric acid concentrations with ranitidine‐antacid was lower than that for corresponding values with placebo in both groups of subjects. The sum of the values for gastric acid concentration with placebo was similar in both groups of subjects, while values with ranitidine‐antacid were higher in subjects with PH than in subjects with NPH.

Thus, subjects with PH differ from subjects with NPH not only in the pattern of heartburn severity following a meal, as illustrated in Figure 1, but also in the time courses of heartburn severity, esophageal acid concentrations, and gastric acid concentrations with different treatments following a meal, as illustrated in Figures 2 and 3.

Because esophageal acid is believed to be the cause of heartburn and gastric acid is believed to be the source of esophageal acid, in the following analyses we examined relationships between values for heartburn severity and corresponding values of esophageal acid concentration as well as for values of esophageal acid concentrations and corresponding values of gastric acid concentrations. We will first analyze results with placebo to examine these relationships in symptomatic GERD subjects and follow up with analyses of results in subjects treated with ranitidine‐antacid to examine how active treatment modifies these relationships.

**FIGURE 4:**
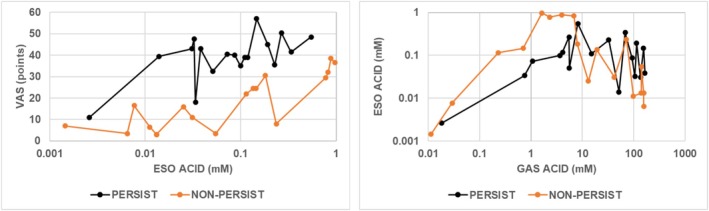
Values with placebo for heartburn severity and mean esophageal acid concentration (Left panel) or mean esophageal acid concentration and mean gastric acid concentration (Right panel). Values given are medians from subjects with PH (*N* = 10) or NPH (*N* = 16). ESO esophageal; GAS gastric; PH persist; NPH non‐persist; VAS visual analog scale.

The left panel in Figure 4 shows that over a similar range of esophageal acid concentrations in subjects treated with placebo, heartburn severity was higher in subjects with PH than in subjects with NPH. This figure also illustrates that the oscillations in esophageal acid concentrations that are illustrated more clearly in Figure 4 right were accompanied by oscillations in heartburn severity. These oscillations are clearer when values for heartburn severity are plotted against the accompanying esophageal acid concentration than when each measure is plotted against time, as shown in Figure 3.

For subjects with PH, an increase in esophageal acid concentration to 0.03 mM was accompanied by an increase in heartburn severity to a VAS value of approximately 40 points. A further increase in esophageal acid concentrations was accompanied by oscillations in both esophageal acid concentrations and heartburn severity (Figure 4 left). These results indicate that the hyperalgesia that occurs at the lowest esophageal acid concentrations oscillated at higher concentrations.

For subjects with NPH, oscillating values for heartburn severity increased progressively with increasing esophageal acid concentrations to the point where the highest esophageal acid concentrations were associated with approximately the same value of heartburn severity as the maximal value in subjects with PH. One can visualize the curve for subjects with NPH in Figure 4 left as representing a rightward shift in the curve for subjects with PH, but with less hyperalgesia.

The right panel in Figure 4 shows that for subjects with PH, esophageal acid concentrations increased progressively to 0.01 mM with gastric acid concentrations up to 4 mM and then oscillated as gastric acid concentrations increased to approximately 150 mM. For subjects with NPH, the magnitude of the increase in esophageal acid concentrations at low gastric acid concentrations was greater than the corresponding increase for subjects with PH. In contrast to results for subjects with PH, as gastric acid concentrations increased from approximately 5 mM to 150 mM in subjects with NPH, esophageal acid concentrations oscillated and also decreased, and this decrease was accompanied by a corresponding oscillating decrease in heartburn severity. Thus, in both phenotypes, esophageal acid concentrations increased during the initial post‐meal period when gastric acid concentrations were increasing but below 5 mM (above pH 3.3), and they oscillated at gastric acid concentrations above 5 mM.

Because subjects in the initial trial (Robinson et al., 2001) chose the time at which they took treatment to relieve heartburn, it was important to compare times of dosing with different treatments to examine the possibility that differences with different treatments might reflect different dose times. The median dose times for subjects with PH and those with NPH were 1.10 h and 0.80 h, respectively, and were not significantly different by Mann–Whitney test (*p* = 0.1619). The median dose times for placebo and ranitidine‐antacid were 1.20 hours and 0.81 hours, respectively, and were significantly different by Wilcoxon Matched Pairs test (*p* = 0.0034). Since both of the median dose times were numerically closest to an assessment time of 1.00 hours, we concluded that a dose time of 1 hour could be used to compare results with ranitidine‐antacid to corresponding results with placebo.

**FIGURE 5:**
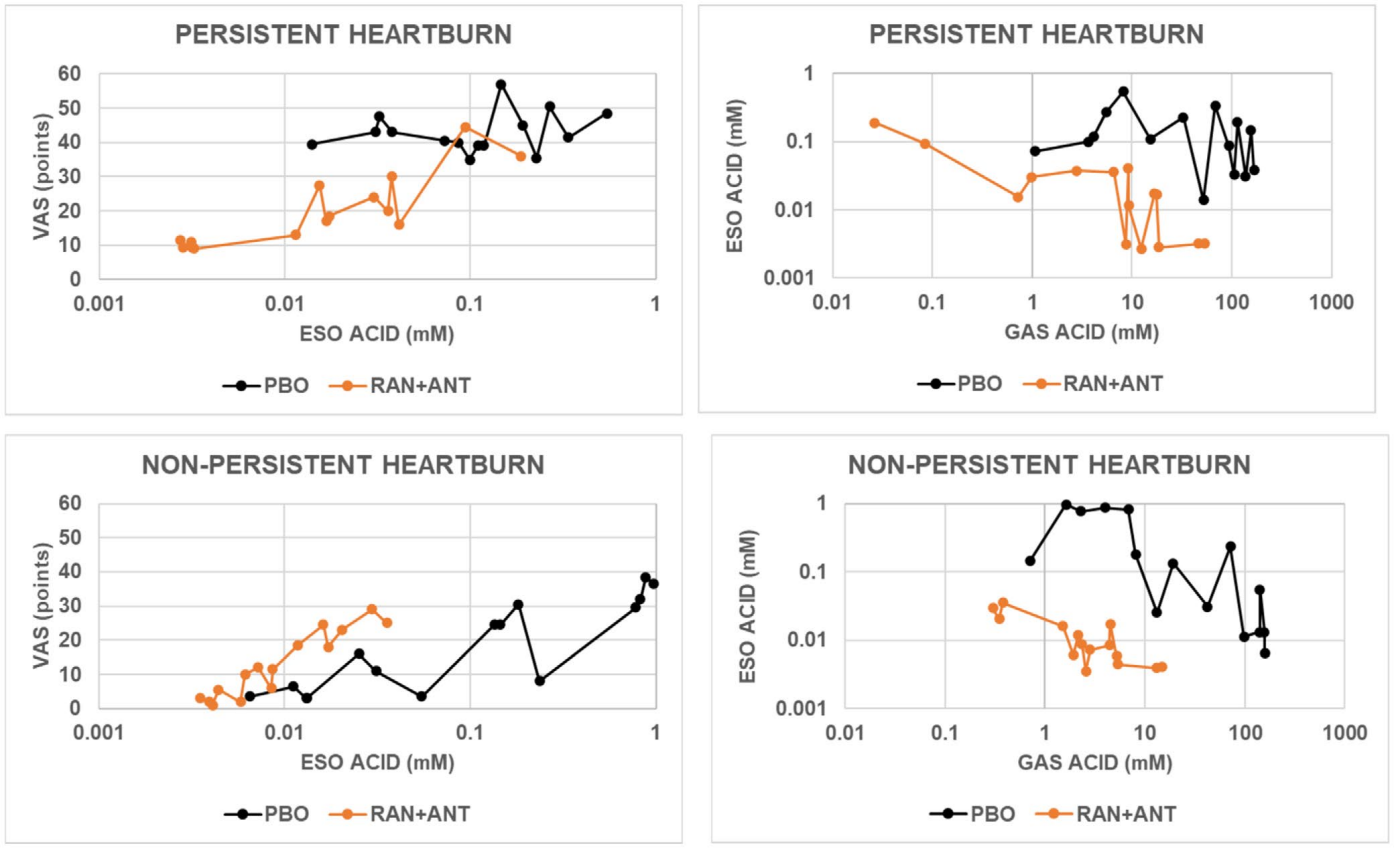
Values for VAS, mean esophageal acid concentration, and mean gastric acid concentration with placebo or ranitidine‐antacid were calculated for each 0.25‐hour interval beginning when treatments were administered 1 h after the end of the meal. Solid symbols are median values for acid concentrations for subjects with PH (*N* = 10) or NPH (*N* = 16). The left panels give observed values for VAS and esophageal acid concentration. The right panels give observed values for esophageal acid concentration and gastric acid concentration. ANT, antacid; ESO, esophageal; GAS, gastric; PBO, placebo; RAN, ranitidine; VAS, visual analog scale.

Figure 5 shows that ranitidine‐antacid decreased overall heartburn severity, esophageal acid concentrations, and gastric acid concentrations in both groups of subjects. It also illustrates the additional information that can be obtained by analyzing results from intervals during the time course.

In subjects with PH, heartburn severity with ranitidine‐antacid at low esophageal acid concentrations was below that with placebo. As esophageal acid concentrations increased, heartburn severity increased progressively to reach a value that was the same as that with placebo. At low gastric acid concentrations in these same subjects, values for esophageal acid concentrations with ranitidine‐antacid were similar to those with placebo. As gastric acid concentrations increased, esophageal acid concentrations decreased progressively. Thus, ranitidine‐antacid decreased gastric acid concentrations, and at the same time increased the accompanying esophageal acid concentrations and heartburn severity. At the lowest value of gastric acid concentrations and the accompanying highest value of esophageal acid concentrations, there was no effect of active treatment on heartburn severity.

The bottom left panel in Figure 5 illustrates that although ranitidine‐antacid decreased overall heartburn severity and overall esophageal acid concentrations in subjects with NPH, values for VAS with ranitidine‐antacid at the lower esophageal acid concentrations were higher than corresponding values with placebo. Thus, ranitidine‐antacid had the unanticipated effect of increasing heartburn severity.

The bottom right panel in Figure 5 illustrates that at all gastric acid concentrations in subjects with NPH, esophageal acid concentrations with ranitidine‐antacid were below those with placebo. As occurred in subjects with PH treated with ranitidine‐antacid, increasing gastric acid concentrations in subjects with NPH were associated with esophageal acid concentrations that decreased progressively with both treatments.

In addition, in subjects with NPH, the slope of the line relating VAS to esophageal acid concentration with ranitidine‐antacid was significantly higher than the corresponding slope with placebo (*p* = 0.0017 by F‐test) indicating that in subjects with NPH, changes in esophageal acid concentrations with ranitidine‐antacid produced significantly greater changes in heartburn severity than comparable changes with placebo. Thus, ranitidine‐antacid decreases esophageal acid exposure, but at the same time, increases esophageal hyperalgesia.

**FIGURE 6:**
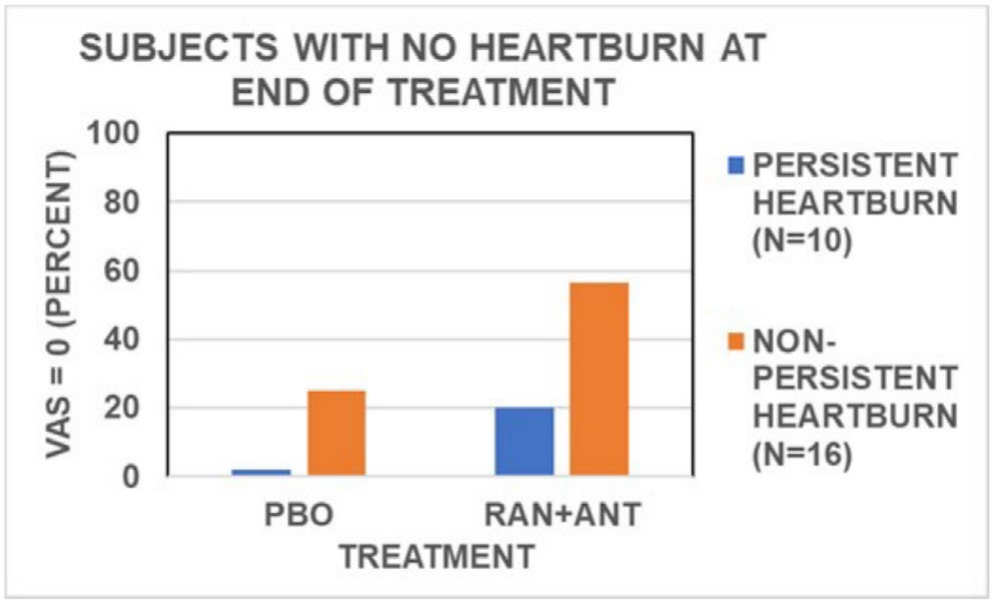
Values for the percentage of subjects with no heartburn at the end of the 4.5‐hour assessment period with placebo or ranitidine‐antacid for subjects with PH (*N* = 10) or NPH (*N* = 16). ANT, antacid; RAN, ranitidine.

Figure 6 displays the percentage of subjects who were free of heartburn 4.5 hours at the end of the standard meal with placebo or ranitidine‐antacid. For subjects with PH, no subject became heartburn‐free with placebo, and 20 percent became heartburn‐free with ranitidine‐antacid. In subjects with PH, the median heartburn severity score was 43 with placebo and decreased to 9 with ranitidine‐antacid. Thus, although treatment with ranitidine plus antacid has only a 1 in 5 chance of making a subject heartburn‐free, there is a good chance that whatever heartburn is experienced is likely to be less severe than that with no active treatment.

Results in Figure 6 from subjects with NPH show that with placebo, 25% became heartburn‐free. Treatment with ranitidine‐antacid increased the heartburn‐free response to 56%. The median heartburn severity score with placebo was 3, and with ranitidine‐antacid was zero. Thus, there was a 56% probability that a subject would become heartburn‐free with ranitidine‐antacid, and a 50% probability that a subject with no treatment would have very mild or no heartburn.

## 5 DISCUSSION

Our analyses identify two distinct clinical phenotypes among symptomatic GERD patients, characterized by different patterns of heartburn severity following a standardized meal. These phenotypes also differ in the relationships among heartburn severity, esophageal acid concentrations, and gastric acid concentrations, as well as their responses to treatment with ranitidine‐antacid. Both phenotypes exhibit esophageal acid‐induced hyperalgesia, which is more pronounced in subjects with PH. This heightened sensitivity may help explain why some symptomatic GERD patients fail to respond to gastric antisecretory therapy.

There were several features of the clinical trial that generated the data for the present analyses that we wish would have been different. Notably, no upper endoscopies were performed, making it impossible to determine which subjects may have had esophagitis or axial hiatal hernia. Additionally, the trial only included subjects with frequent heartburn. While heartburn frequency is a common inclusion criterion for GERD trials, the applicability of these findings to GERD subjects with other symptoms, such as regurgitation or chest pain, remains uncertain.

Another limitation was the variation in meal composition and quantity across subjects. For example, one participant might have ingested 1 biscuit, 2 chocolate milks, and 1 patty, while another might have consumed 2 biscuits, 1 chocolate milk, and 2 patties. Although each meal was homogenized and titrated to pH 2 for the calculation of meal‐stimulated gastric acid secretion, we used the acid required for titration as a surrogate for meal size. This approach showed no significant difference in meal size between the two groups of subjects. However, it is possible that differences in specific meal constituents may have influenced the time courses of heartburn severity and esophageal acid concentration. Furthermore, dosing times were not fixed, and subjects self‐administered treatment based on their heartburn severity. A significant difference was observed in the dosing time for placebo versus ranitidine‐antacid. However, this difference did not appear to account for the observed differences in the time courses of heartburn severity, esophageal acid concentrations, or gastric acid concentrations. The lack of blinding to the antacid could have influenced dose timing and subjective ratings of heartburn severity, potentially contributing to differences within groups, although not between groups. Ideally, a fixed dosing time, standardized meals, and blinded antacid administration would have improved the usefulness of the clinical study for our present analyses.

Our present analyses of heartburn severity following a standard meal identified two distinct clinical phenotypes of symptomatic GERD – PH severity where heartburn severity increases to a maximum and remains elevated, and NPH severity where heartburn severity increases to a maximum and then decreases toward baseline. Identification of these phenotypes requires no special equipment other than a standard meal and a mechanism for recording heartburn severity on a visual analog scale at regular intervals. Values of heartburn severity following a standard meal in an individual subject can be used to assign that subject to one of the clinical phenotypes. Measures of the time courses of heartburn severity, esophageal acid, and gastric acid with different treatments can identify significant differences between the two phenotypes.

An important advantage of the phenotypes based on heartburn severity is that they are based on symptoms—a major reason that GERD patients seek healthcare. In addition, using a standard meal and standard assessments means that patient responses, including before and after treatment, can be compared under the same clinical conditions. Other advantages illustrated by the present analyses are that it is possible to assess a patient's symptom response to treatment, and that the pathophysiology of GERD symptoms can be explored easily by adding measurements of esophageal and gastric pH.

The Lyon Consensus (Ghisa et al., 2020; Gyawali et al., 2018) identified three different phenotypes of symptomatic GERD based on the time esophageal pH is below 4 and whether symptoms are associated with esophageal reflux episodes identified initially by a decrease in esophageal pH to below pH 4 or more recently, by a change in esophageal impedance (Kamal et al., 2020). These phenotypes do not consider possible relationships between symptoms and esophageal acid exposure or gastric acidity, and the Consensus commented that the association of esophageal acid exposure and symptoms of GERD is weak. In addition, the measurements of esophageal pH and impedance have usually occurred in a setting where physical activity, beginning time of measurements, and food and drink intake are not standardized.

The present analyses illustrate that time is an important variable, whereas the Lyon Consensus does not appear to have considered time as being potentially related to any of the measurements (Ghisa et al., 2020; Gyawali et al., 2018). Nearly all published literature in the GERD field specifies or implies a direct relationship between esophageal acid and symptoms. For example, the occurrence of GERD symptoms has been reported to be associated with increased esophageal acid exposure during the time before a symptom (Beedassy et al., 2000; Bredenoord et al., 2006) and reductions in esophageal acidity with gastric antisecretory agents can reduce the occurrence of symptoms (Allgood et al., 2005). Previous analyses, however, found an inverse relationship between the probability of a symptom and the value of esophageal acid exposure that preceded the symptom in each of the three symptomatic GERD phenotypes described by the Lyon Consensus (Gardner, 2022a, 2022b). Moreover, esophageal acid sensitivity oscillated during the course of a 24‐h assessment in each phenotype. These findings indicate that it is unlikely that values for total esophageal acid exposure will provide meaningful information regarding symptom frequency in symptomatic GERD subjects because some symptoms will be preceded by low values of esophageal acid, while other symptoms will be preceded by high values of esophageal acid. Our present results might also result from the meal “fixing” esophageal acid sensitivity in a high‐sensitive state in PH subjects but not in NPH subjects.

We found that in subjects treated with placebo, the time course of heartburn severity was significantly higher in subjects with PH than in subjects with NPH. In contrast, the time course of esophageal acid concentration was significantly higher in subjects with NPH than in subjects with PH. These findings indicate that in subjects treated with placebo, the sequence of individual values of VAS and esophageal acid concentration is important in distinguishing subjects with PH from those with NPH, and that overall, subjects with PH have higher esophageal hyperalgesia than subjects with NPH.

We also found clear differences between subjects with PH and subjects with NPH in the time courses for heartburn severity, esophageal acid concentration, and gastric acid concentration with placebo as well as with ranitidine‐antacid. These results provide additional support for the existence of two distinct phenotypes of subjects with symptomatic GERD. It is difficult to attribute these differences to the variations in dose times, meal size, the lack of blinding of the antacid, or the lack of information from an upper endoscopy. On the other hand, if all subjects with PH and none with NPH had erosive esophagitis, this could provide useful information for clinicians as well as for the development of agents to treat heartburn in subjects with PH.

In the present analyses, in PH subjects, 56% and 89% of esophageal acid concentrations were above pH 4.0 with placebo and ranitidine‐antacid, respectively, while in NPH subjects, 50% and 100% of esophageal acid concentrations were above pH 4 with placebo and ranitidine‐antacid, respectively. Except for results with placebo in subjects with PH, the esophageal acid concentrations above pH 4 and the associated values for heartburn severity decreased in parallel during the last hours of the post‐meal assessment period. Thus, as demonstrated previously (Gardner, 2022a, 2022b), low esophageal acid exposure is a common companion of heartburn in symptomatic GERD subjects, and this feature can impact the efficacy of treatments designed to prevent or relieve GERD symptoms.

Another surprising feature of the decreases in esophageal acid exposure and heartburn severity was their association with progressive increases in gastric acid concentrations. This paradoxical relationship between esophageal acid concentrations and gastric acid concentrations illustrates why it can be important to examine time courses for these relationships. The knowledge that gastric acid is the source of esophageal acid and the findings that agents that inhibit gastric acid secretion also reduce esophageal acid exposure would lead one to believe that increasing gastric acid concentrations after a meal would be accompanied by increasing esophageal acid concentrations instead of the observed inverse relationship.

One possible explanation for the lack of agreement between the time course for esophageal acid concentrations and that for gastric acid concentrations is that the gastric pH probe does not measure the acid that is the source of acid reflux into the esophagus. Others have reported that following a meal, acid that refluxes into the esophagus originates from a gastric “acid pocket” that sits on top of the ingested meal and can persist for at least 48 min (Fletcher et al., 2001; Kahrilas et al., 2013). Such a phenomenon could result in esophageal acid originating from a source that is not measured by the gastric pH electrode, typically residing in the proximal gastric body. It also seems possible that changes in gastric volume, which were not measured in the present study, might be an important determinant of esophageal acid concentrations. Nevertheless, regardless of the explanation for the relationships between esophageal and gastric acid concentrations, our results show clearly that treating subjects with symptomatic GERD with ranitidine‐antacid following a standard meal can result in increased heartburn severity. These findings could also be important clinically when considering the extent to which esophageal acid has to be decreased to reduce heartburn severity in symptomatic GERD subjects, and the extent to which gastric acid has to be decreased to decrease esophageal acid.

The present results are also relevant to the issue of GERD subjects who fail to respond to gastric antisecretory therapy (DeBortoli et al., 2014). Most published studies appear to have attempted to answer the question “Given a failed response to therapy, what is the probability that a subject has GERD?” The present results, however, can be viewed as attempting to answer the question “Given that a subject has GERD, what is the probability of a failed response to therapy?”. Most published studies of suspected GERD subjects who fail treatment, have measured esophageal pH and esophageal impedance, but have only evaluated a possible association of symptoms and reflux episodes even though it is possible to measure esophageal acid exposure that is associated with each symptom that occurs during the procedure (Gardner, 2022a, 2022b). In addition, these assessments have been performed after treatment has been stopped for several days.

Finally, our present results illustrate two different mechanisms that might lead to a failed response to GERD treatment.

First, in all conditions except for subjects with PH treated with placebo, treatment resulted in esophageal acid concentrations as low as pH 5. Under such conditions, an increase in esophageal acid concentration, such as might occur with a transient reflux episode after a meal or an acidic drink (table‐beverage‐acidity.pdf, n.d.; Feldman & Barnett, 1995; Johnson et al., 2010), might cause heartburn or increase heartburn severity. Insofar as we are aware, investigators who evaluate suspected GERD subjects who fail treatment have not tested an acidic drink, although they have taken a history of GERD symptoms occurring with acidic drinks.

Second, in subjects with NPH treated with ranitidine‐antacid, nearly all values for heartburn severity are higher than corresponding values for heartburn severity when the same subjects were treated with placebo. It may be that in subjects with NPH treated with placebo, the higher values for esophageal acid exposure that occur early in the post‐meal period cause desensitization to subsequent esophageal acid exposure. Treating these same subjects with ranitidine‐antacid avoids this desensitization and leads to greater heartburn severity with the subsequent low esophageal acid exposure.

## FUNDING INFORMATION

No external funding.

## CONFLICT OF INTEREST STATEMENT

Neither Dr. Gardner nor Dr. Triadafilopoulos has a conflict of interest with the analyses in the present paper.

## Data Availability

Processed data used for figures and tables are available from the corresponding author upon reasonable request. Raw data are not shared due to limitations in data storage and anonymization. The work described in this manuscript has not been published, is not being considered for publication elsewhere, and has not been posted on a preprint server.
